# The relationship between negative emotions and adjustment disorder in young adults: the mediating role of rumination and insomnia

**DOI:** 10.3389/fpsyt.2025.1474108

**Published:** 2025-02-24

**Authors:** Xinyue Shao, Zhao Dong, Shuai Zhang, Yunyun Qiao, Hongwei Zhang, Hua Guo

**Affiliations:** Department of Child and Adolescent Psychology Division I, The Affiliated Encephalopathy Hospital of Zhengzhou University, Zhumadian, China

**Keywords:** negative emotions, adjustment disorder, rumination, insomnia, multiple mediating role, young adults

## Abstract

**Introduction:**

This study aimed to investigate the influence of negative emotions on adjustment disorder (AjD) in young adults, focusing on the mediating role of rumination and insomnia.

**Methods:**

The study recruited 2015 young patients (aged 18–35) receiving treatment at the Psychosomatic Medicine Department of the Affiliated Encephalopathy Hospital of Zhengzhou University from February 2023 to March 2024. Participants completed the Depression Anxiety and Stress Scale (DASS), Ruminative Responses Scale (RRS), Insomnia Severity Scale (ISI), and Adjustment Disorder – New Module 20(ADNM-20) to assess negative emotions, rumination thinking, sleep status, and AjD. Data were analyzed utilizing descriptive statistics, correlation, hierarchical linear regression, and mediation analyses.

**Results:**

1) AjD was significantly influenced by being an only child and family composition, but there was no significant gender difference. Scores for negative emotions, rumination, insomnia, and AjD varied significantly different among different age groups, with the 30–35 age group scoring significantly higher than others. 2) Total scores for the DASS-21and its subscales, the RRS and its subscales, insomnia, and AjD were significantly correlated (*p* < 0.01). Negative emotions and ruminative thoughts predicted AjD, accounting for 47.8% of the total variation in AjD. 3) Negative emotions positively predicted AjD (β = 0.37, *p* < 0.001). Negative emotions affect AjD in young adults through rumination and insomnia alone and together, and the mediating effect accounts for 34%, 7.9%, and 20% of the total effect.

**Discussion:**

The study’s findings suggest that rumination and insomnia play significant mediating roles in the relationship between negative emotions and AjD in young adults. Negative emotions directly affect AjD and have indirect effects through rumination and insomnia.

## 1 Introduction

Adjustment disorder (AjD) is a reaction caused by important life changes or stressful events, characterized primarily by emotional disorders accompanied by maladaptive behaviors or physiological dysfunction, considerably affecting social functioning (1, 2). According to the World Health Organization (WHO) International Classification of Diseases 11^th^ version, AjD is defined as the development of emotional and behavioral symptoms in response to external life stressors (3). AjD is one of the mental disorders most commonly diagnosed in clinical practice that includes major emotional or behavioral symptoms that generally result from major life events or other stressors, such as serious illness (4). Epidemiological studies have demonstrated that the prevalence of AjD is 2% in the general population (5), 27% among individuals who have recently experienced involuntary job loss (6), and 18% among widowed individuals (7). AjD is particularly prevalent in counseling and liaison settings (8). In psychiatric consultations, 12% of cases involve patients diagnosed with AjD, with an additional 11% considered probable cases (9). According to statistics, the population of young adults aged 14-35 in China is about 400 million, accounting for 28.4% of the total population of the country. In a cross-sectional study (10), Yousif and colleagues found that the prevalence of AjD in the younger age group (15-33 years old) was significantly higher than that in other age groups. Some studies have also found that AjD with depressed mood are the most common diagnoses of suicide attempts in young people (11, 12). Given the high incidence and suicide rates of AjD in young adults, there is a need for greater understanding and awareness of the pathophysiological and psychological processes involved, which are essential for the prevention and treatment of AjD in young adults.

AjD is primarily associated with susceptibility and maladjustment. For related and risk factors, it has been reported that the likelihood of AjD varies according to the type and impact of stressors, including personal factors such as sex (13), age (14), self-efficacy, and coping, as well as interpersonal factors such as economic status (10) and social support (15). The core symptoms—adaptive disorder focus, rumination, and worry—can be classified as factual thinking related to stressors that are often associated with negative emotions (16). Negative emotions are a crucial component of subjective perspectives and reflect personal experiences of subjective tension and unpleasant involvement. Depression and anxiety are common negative emotions experienced by young adults. Long-term immersion in depression, anxiety, pressure, and other negative emotions will not only reduce general enthusiasm and initiative but also have a negative impact on individuals, resulting in social dysfunction such as AjD, as well as self-harm and suicide in severe cases (17, 18). It has been found that negative emotion is one of the risk factors for adverse reactions to AjD, and thus the pathopsychological process from negative emotion to AjD is worth investigating.

Prior studies have found that rumination is related to the occurrence, development, and persistence of negative emotions (19–21); it has been defined as “after encountering negative life events, the individual’s thinking stays under the influence of life events and repeatedly thinks about the causes, consequences and feelings of the events” (20). Ruminative thinking is a maladaptive cognitive style that causes individuals to fall into a vicious cycle of negative emotions and cognition under external pressures and cognitive dissonance. Individuals who frequently experience stressful events are more inclined to have more serious and long-term negative emotions post-event and are therefore more likely to engage in ruminative thinking (22, 23). Numerous studies have demonstrated that rumination is an important factor that influences negative emotions and that there is a significant positive correlation between rumination and negative emotions. According to the reaction style theory, rumination not only triggers and prolongs negative emotions, such as depression and anxiety (24), but also aggravates negative emotions and negative cognition (25). More recently, evidence from an empirical sampling study suggested that rumination engagement partially mediates the link between negative events and negative emotions (26). Emotional regulation of cognitive dissonance has been emphasized as a key factor in the occurrence and maintenance of negative emotions.

Insomnia refers to the continuous struggle to fall asleep, destruction of sleep integrity, and a decline in sleep quality, often accompanied by daytime dysfunction despite adequate sleep opportunities and a conducive sleeping environment (27). Insomnia is a disease (primary insomnia) and a symptom of other diseases (comorbid insomnia) (28). Maladjustment is one of the three core symptom factors of the negative event stress response, and its symptoms often interfere with daily functioning, such as difficulty concentrating or sleep disturbance (2). Studies have found a close relationship between negative emotions and insomnia, whereby insomnia plays a causal role in the development of negative emotions such as depression and anxiety (29). Sleep disorders are common among patients with depression (30); the higher the depressive mood, the worse the sleep quality (31, 32). It is often associated with anxiety disorders (29, 33). Sleep quality often interacts with emotional disorders, and sleep-wake regulation disorders aggravate negative emotions, forming a vicious cycle that may lead to inevitable negative thoughts and behaviors (34). Additionally, the influence of rumination on insomnia has been confirmed (35–37). The cognitive model of sleep posits that rumination, a relatively common intrusive thought, can prevent individuals from entering and maintaining sleep activities (28). Studies have found that the higher the level of rumination thinking, the worse the quality of sleep (35–37). As an important indicator of mental health, sleep is closely related to the mental state of the individual (38) and has a substantial impact on the occurrence, development, and maintenance of emotional problems in young individuals (39). In summary, there is a paired correlation between negative emotions, rumination, and insomnia. However, research on the internal relationship between rumination and insomnia in young individuals with AjD needs to be enhanced, and that between rumination, insomnia, negative emotions, and AjD warrants further research.

Based on the findings of prior studies and the theory of response styles, this study developed a mediation model to explore the relationships and combined effects among negative emotions, rumination, and insomnia in young patients with AjD. The study considered negative emotions, rumination, and insomnia as independent variables, with rumination and insomnia as mediating variables and AjD as the dependent variable. Additionally, the study sought to determine whether rumination can induce, prolong, or exacerbate negative emotions, leading to insomnia and ultimately resulting in AjD. This research aimed to reveal the mechanisms by which negative emotions contribute to AjD and to elucidate the mediating roles of rumination and insomnia.

## 2 Methods

### 2.1 Participants

Baseline assessment was conducted from February 2023 to March 2024. Participants with negative emotional problems, whose ages ranged from 18 to 44 years old and were treated in the psychosomatic outpatient department, were recruited consecutively through the Affiliated Encephalopathy Hospital of Zhengzhou University (Zhumadian Second People’s Hospital), most of whom came from Zhumadian and the surrounding areas. Participants were excluded if they were below 18 years of age; were not able to participate as a result of severe physical diseases, severe mental disorders, serious disabilities; or if the basic information was incomplete and could not be verified and supplemented by other means.

A total of 2176 questionnaires were collected, of which 161 were excluded for not meeting the inclusion criteria and for providing answers that were too short, a response rate of 92.60%. Among the 2015 participants, 55.3% (*n* =1115) were male and 44.7% (*n* = 900) female. The mean age of the sample was 25.41 years (SD = 3.91, range 18–78 years). Among the participants, 92.0% (*n* =1856) of participants reported living in two-parent family, while 46.9% (*n* =946) were only child. The study was approved by the Affiliated Encephalopathy Hospital of Zhengzhou University (ethics approval number: KS-2023-005-01).

### 2.2 Measures

#### 2.2.1 General information questionnaire

The initial part of the survey was a self-developed general information questionnaire that included the measurement of sociodemographic variables such as gender, age, being an only child, and family composition.

#### 2.2.2 Depression anxiety and stress scale

The Depression Anxiety and Stress Scale (DASS) was originally compiled by Lovibond in 1995 (40). This study adopted a simplified version of the DASS (DASS-21) revised by Chinese scholar Gong Xiang (41). The full scale contains 21 items, and the three subscales (depression, anxiety, and stress) each contain seven items. All subscales are scored from 0 (“inconsistent”) to 3 (“always consistent”); in the dimensions of depression, anxiety, and stress, higher scores on the survey indicate a more severe level of these negative emotions.The internal consistency coefficient of the scale in this study exhibited a Cronbach’s α of 0.940, and the Cronbach’s *a* coefficients for the depression, anxiety, and stress subscales are 0.794, 0.746, and 0.752 respectively, indicating good reliability and validity of the scale.

#### 2.2.3 Ruminative Responses Scale

The Ruminative Responses Scale (RRS) was compiled by Nolen-Hoeksema and is used to assess individual differences in the tendency to ruminate. It has high internal consistency as well as acceptable convergent validity (19), and its Chinese version was revised by Han and Yang (2009) (42). The scale consists of 22 items divided into three categories: rumination, brooding, and reflective pondering. It is scored from 1 “never” to 4 “always,” and the higher the score, the more severe the rumination.

#### 2.2.4 Insomnia Severity Index

The Insomnia Severity Index (ISI) was compiled by Morin (43) to assess the nature, severity, and impact of insomnia., with seven items and a 5-level score ranging from 0 to 4 points. The dimensions evaluated are the severity of sleep onset, sleep maintenance, early morning awakening issues, sleep dissatisfaction, interference of sleep difficulties with daytime functioning, noticeability of sleep issues by others, and distress caused by sleep challenges. The higher the total score on the scale, the more severe the insomnia. Cronbach’s *a* for the total score in this study was 0.84, indicating good reliability and validity.

#### 2.2.5 Adjustment disorder – New module 20

The Adjustment Disorder – New Module 20 (ADNM-20) is a self-report measurement developed by Glaesmer to assess whether individuals have AjD and to measure the symptoms of AjD (5). The scale has 20 items, including six factors. Pre-occupation (entries 2, 4, 13, 15) and failure to adapt (entries 10, 17, 19, 20) were the two core symptom clusters. Additionally, four accessory symptom factors were included: anxiety (item 6, 16), depressive mood (item 1, 5, 18), impulsivity (item 8, 9, 12), and avoidance (item 3, 7, 11, 14). Item 20 was a functional impairment item. Participants indicated the frequency of all symptoms assessed utilizing these items on a 4-point Likert scale (1 = “never” to 4 = “often”); a sum score over all items measures the severity of AjD symptoms. In this study, ADNM- 20 scores had an excellent internal consistency of α = 0.91.

### 2.3 Procedure

The survey was conducted in The Affiliated Encephalopathy Hospital of Zhengzhou University, and the electronic questionnaire link was distributed through the questionnaire star platform. All participants were presented with an informed consent sheet, the contents of which were explained by a trained research assistant. The participants were assured of the confidentiality of their responses and the voluntary nature of their participation.

An anonymous assessment was adopted, and the purpose of the study and relevant requirements for the answers were clarified to participants utilizing standardized guidelines; the entire process took approximately 20 to 30 minutes. After all participants completed the questionnaire, they were validated by two trained psychologists. Questionnaires were regarded as invalid and were excluded if they had missing items, unusually short response times, or irregularities in the response options.

The collected data included sociodemographic characteristics, emotional states (such as anxiety, depression, and stress symptoms), sleep patterns, and AjD.

### 2.4 Statistical analysis

Descriptive statistics were calculated from basic demographic data. Statistical analysis was conducted with IBM SPSS Statistics 28. Pearson’s correlation analysis was utilized to examine the correlation between the scores of each scale and the scores of its sub-dimensions. A univariate test was conducted utilizing the t-test, ANOVA and *post hoc* test with a statistical significance of p < 0.05. Multiple regression was utilized to identify risk factors for the multivariate analysis. The regression model uses adjustment *R²* to explains the variance. Amos 7.0 statistical software was used to conduct a path analysis of potential variables in the structural model to determine the mediating effect of rumination and insomnia on negative emotions and AjD. The significance of the mediating effect was tested utilizing a bias-corrected bootstrap method.

## 3 Results

### 3.1 Demographics of participants

An independent samples t-test, ANOVA and *post hoc* test were conducted to assess demographic differences in negative emotions, rumination, insomnia, and AjD among young individuals. Descriptive statistics for all sociodemographic variables included in the study are presented in **Table 1**. There were no significant differences in the total mean scores of negative emotions, rumination, and insomnia based on demographic variables such as gender and family completeness (*p* > 0.05). However, regarding AjD, the scores for only child were significantly higher than those for non-only child, and those for individuals from two-parent family were significantly higher than those from a single parent family, with no significant difference between genders. Scores of negative emotions, rumination, insomnia, and AjD varied significantly across different age groups. Post-test results showed that the score of AjD in the age group of 30 to 35-year-olds was significantly higher than that in other age groups(LSD-t=2.189-8.119,P< 0.05).

**Table 1:** Scores of various scales in in young adults of different demographic characteristics.

Variable	DASS-21	RRS	ISI	ADNM-20
Men (*n* = 1115)	4.20±6.46	27.12±6.91	3.13±4.02	24.35±8.26
Women (*n* = 900)	4.15±6.01	27.50±7.47	3.19±4.24	24.31±8.35
t	0.183	-1.200	-0.318	0.091
p	0.269	0.135	0.205	0.998
only child (*n* = 946)	4.41±6.65	27.62±7.62	3.24±4.22	24.96±8.99
non-only child (*n* = 1069)	3.97±5.88	27.00±6.73	3.08±4.02	23.78±7.59
t	1.571	1.929	0.879	3.202
p	0.004	0.007	0.104	<0.001
two-parent family (*n* = 1854)	4.19±6.29	27.27±7.08	3.13±4.08	24.39±8.43
a single parent family (*n* = 161)	4.04±5.87	27.53±8.11	3.40±4.54	23.64±6.48
*t*	0.303	-0.435	-0.778	1.102
*p*	0.785	0.360	0.171	0.049
15–19 (*n* = 15)	3.53±3.89	27.40±5.03	1.73±2.15	22.60±5.23
20–24 (*n*=1014)	3.40±5.49	26.87±6.78	2.82±3.75	23.19±6.40
25–29 (*n*=640)	4.11±5.62	27.01±6.56	3.07±3.80	24.57±8.01
30–35 (*n*=346)	6.62±8.56	29.04±8.94	4.36±5.39	27.32±12.17
*F*	23.728	8.487	13.124	22.445
*p*	< 0.001	< 0.001	< 0.001	< 0.001

### 3.2 Correlation analysis of negative emotions, rumination, insomnia, and AjD

As shown in **Table 2**, the Pearson product-difference correlation analysis demonstrated all factors in the model were significantly and positively correlated, ranging from 0.445 to 0.952. (*p* < 0.01). The correlation coefficients between AjD symptoms and depression, anxiety, as well as stress, were *r* = 0.615 (*p* < 0.001), *r* = 0.572 (*p* < 0.001), and *r* = 0.600 (*p* < 0.001), respectively.

**Table 2:** Pearson correlations among the study variables.

	1	2	3	4	5	6	7	8	9	10	11	12	13	14	15	16
1. Depression	1															
2. Anxiety	0.810^**^	1														
3. Stress	0.810^**^	0.821^**^	1													
4. DASS-21	0.925^**^	0.927^**^	0.952^**^	1												
5. Symptom rumination	0.730^**^	0.680^**^	0.683^**^	0.744^**^	1											
6. Brooding	0.529^**^	0.532^**^	0.575^**^	0.586^**^	0.792^**^	1										
7. Reflective pondering	0.512^**^	0.512^**^	0.532^**^	0.555^**^	0.783^**^	0.814^**^	1									
8. PRS	0.667^**^	0.642^**^	0.661^**^	0.702^**^	0.954^**^	0.916^**^	0.906^**^	1								
9. ISI	0.582^**^	0.588^**^	0.593^**^	0.628^**^	0.584^**^	0.471^**^	0.457^**^	0.560^**^	1							
10. Preoccupations with the stressor	0.594^**^	0.556^**^	0.583^**^	0.618^**^	0.637^**^	0.515^**^	0.494^**^	0.610^**^	0.500^**^	1						
11. Failure to adapt	0.576^**^	0.526^**^	0.535^**^	0.582^**^	0.605^**^	0.458^**^	0.445^**^	0.564^**^	0.477^**^	0.839^**^	1					
12. Avoidance	0.513^**^	0.493^**^	0.522^**^	0.546^**^	0.564^**^	0.501^**^	0.491^**^	0.567^**^	0.432^**^	0.808^**^	0.750^**^	1				
13. Depressed mood	0.578^**^	0.522^**^	0.545^**^	0.586^**^	0.618^**^	0.501^**^	0.493^**^	0.595^**^	0.473^**^	0.816^**^	0.793^**^	0.746^**^	1			
14. Anxiety	0.585^**^	0.532^**^	0.560^**^	0.597^**^	0.622^**^	0.492^**^	0.481^**^	0.592^**^	0.477^**^	0.876^**^	0.855^**^	0.798^**^	0.796^**^	1		
15. Impulse disturbance	0.552^**^	0.520^**^	0.555^**^	0.581^**^	0.594^**^	0.464^**^	0.452^**^	0.562^**^	0.476^**^	0.846^**^	0.848^**^	0.770^**^	0.759^**^	0.822^**^	1	
16. ADMN-20	0.615^**^	0.572^**^	0.600^**^	0.637^**^	0.660^**^	0.535^**^	0.520^**^	0.634^**^	0.514^**^	0.947^**^	0.921^**^	0.899^**^	0.886^**^	0.926^**^	0.915^**^	1

Cronbach's alpha values are listed diagonally. ** *p* < 0.01 (two-tailed).

### 3.3 Structural model analyses

The relationship between negative emotions, rumination, insomnia, and AjD was explored. To exclude the interaction between variables and test the predictive effect of each variable on adaptation disorders, a multilevel regression analysis was utilized. The regression analysis comprised three models. Model 1 included anxiety, stress, and depression on the DASS-21. Model 2 included anxiety, stress, depression, and rumination. Model 3 included anxiety, stress, depression, rumination, and insomnia. The dependent variable was adjustment barrier.

As exhibited in **Table 3**, the adjusted *R²* value of Model 1 was 0.410, indicating that negative emotions could explain 41.0% of the variance in adjusted disorders. When rumination was included in Model 1, adjusted *R* ^2^ changed from 0.410 to 0.477, indicating that negative emotions and rumination explained 47.7% of the AjD. The coefficient of ruminative thought was 0.422, which was significant (*t* = 16.065, *p* < 0.001) and had a positive effect on AjD. When insomnia was included to Model 3, adjusted *R* ^2^ increased from 0.477 to 0.486, indicating that insomnia could explain 0.9% of AjD based on Model 2. The regression coefficient of insomnia was 0.249, which was significant (*p* < 0.001), indicating that rumination had a significant positive effect on insomnia. In Model 1, anxiety, stress, and depression made independent positive contributions to AjD. Furthermore, when rumination and insomnia were included in Model 3, the contributions of depression, anxiety, and stress changed. However, anxiety was not significant.

**Table 3:** Hierarchical regression analysis predicting adjustment disorder.

	Model 1					Model 2					Model 3				
	B	SE	β	t	p	B	SE	β	t	p	B	SE	β	t	p
Depression	1.387	0.133	0.337	10.409	<0.001	0.919	0.129	0.223	7.135	<0.001	0.853	0.128	0.207	6.649	<0.001
Anxiety	0.42	0.146	0.096	2.886	0.004	0.158	0.138	0.036	1.144	0.253	0.06	0.138	0.014	0.435	0.663
Stress	0.743	0.099	0.248	7.474	<0.001	0.445	0.095	0.149	4.658	<0.001	0.377	0.095	0.126	3.952	<0.001
Rumination						0.422	0.026	0.364	16.065	<0.001	0.388	0.027	0.335	14.555	<0.001
Insomnia											0.249	0.042	0.124	5.877	<0.001
Adjusted R²			0.410					0.477					0.486		
R^2^			0.411					0.478					0.487		
△R^2^								0.063					0.009		
F	F (3,2011) = 467.760					F (4,2010) = 460.190					F (5,2009) = 381.203				
p	<0.001					<0.001					<0.001				

B, Unstandardized regression weight; SE, Standard error for B; β, Standardized beta weight; *p*, statistical significance.

(*n* = 2015).

This study found that increased rumination and insomnia significantly predicted AjD. This model accounted for half of the variance in the changes in AjD (*F* (5,2009) = 381.203, *p* < 0.001; adjusted *R* ^2^ = 0.486). Regression models identified depression, stress, rumination, and insomnia as important predictors of AjD.

**Figure 1:**
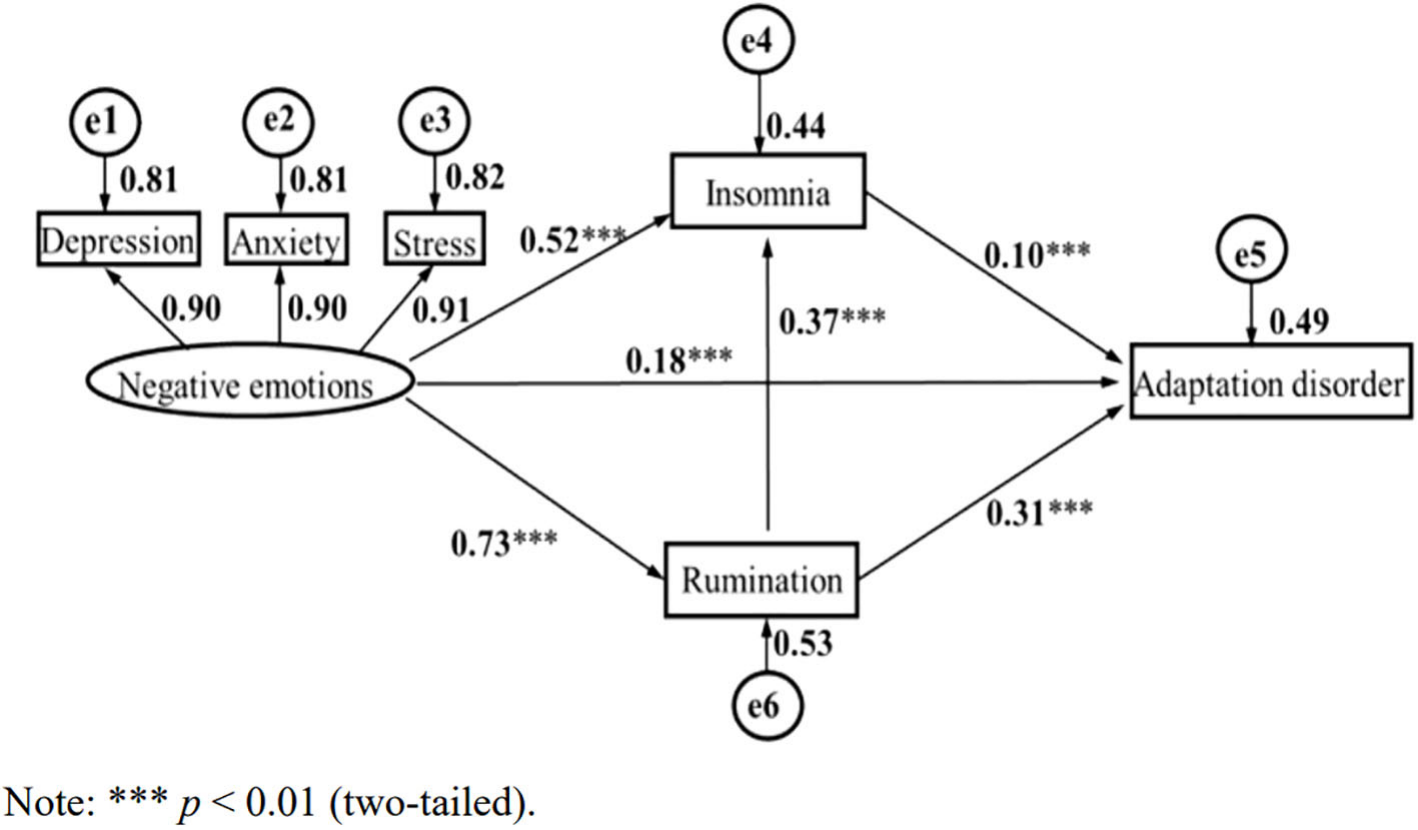
Negative emotion, rumination, insomnia, and adaptation disorder chain mediation effect. ***p<0.01 (two-tailed).

These results provide preliminary support for the proposed structural equation model exhibited in **Figure 1**. Maximum likelihood estimation was employed to calculate the goodness-of-fit indices (χ^2^/df = 25.597/6 = 4.266, GFI = 0.996, AGFI = 0.985, CFI =0.998, TLI = 0.994, NFI = 0.994, RMSEA =0.040).

According to existing studies and the above findings, to further explore the specific role of ruminative thinking and insomnia in negative emotions and AjD, a structural equation model was utilized to analyze the influence of negative emotions on AjD and to assess the mediating effect of ruminative thinking and insomnia. The structural equation model was constructed with negative emotions as an exogenous variable, rumination and insomnia as endogenous latent variables, adaptation disorder as the dependent variable, and depression, anxiety, and stress as negative emotion control variables. As shown in **Figure 1**, in the measurement model, the standardized load of negative emotions on the latent variable was above 0.90, which indicates that the measurement of the latent variable by the observed variable was sufficient and effective.

**Figure 1** demonstrates that negative emotion can significantly and positively predict AjD (β = 0.37, *p* < 0.001), insomnia (β = 0.52, *p* < 0.001), as well as rumination (β = 0.73, *p* < 0.001). Rumination was a significant positive predictor of adaptation disorder (β = 0.31, *p* < 0.001) and insomnia (β = 0.18, *p* < 0.001). Insomnia was a positive predictor of AjD (β = 0.10, *p* < 0.001).

Bootstrapping techniques were utilized to evaluate multiple mediations. As exhibited in **Table 4**, all paths were explicitly significant (zero was not included in the 95% CI), indicating that ruminative thinking and insomnia had mediating effects on negative emotions and AjD.

**Table 4:** Bootstrap test of the mediating effect of rumination and insomnia on negative emotion and AjD.

Path	Estimate	Effective size	SE	Bias-Corrected 95% CI	
				Lower	Upper
Negative emotions→ rumination→AjD	1.028	34	0.131	0.781	1.290
Negative emotions→ insomnia→AjD	0.238	7.9	0.072	0.104	0.391
Negative emotions→ rumination→insomnia→AjD	0.060	20	0.024	0.022	0.118

→ indicates the direction of the mediation effect.

In this model, the direct effect of negative emotions on AjD was 0.37, and the total mediation effect was 1.326 (1.028 + 0.238 + 0.060). The results indicate that negative emotions can not only directly predict suicidal ideation but can also predict AjD through the chain-mediating effect of rumination and insomnia, that is, rumination and insomnia play a chain-mediating role between negative emotion and AjD in young individuals. Integrating **Figure 1** with **Table 5**, a clearer association can be observed where rumination and insomnia exert multiple mediation effects on the relationship between negative emotions and AjD.

**Table 5:** Fit index of the mediation model.

Index	χ^2^	df	χ^2^/df	GFI	AGFI	CFI	TLI	NFI	RMSEA
Value	25.597	6	4.266	0.996	0.985	0.998	0.994	0.994	0.040

GFI, goodness-of-fit index; AGFI, adjusted goodness-of-fit index; CFI, comparative fit index; TLI, Tucker–Lewis index; NFI, normed fit index; RMSEA, root mean square error of approximation.

## 4 Discussion

This study found that, in terms of AjD, the scores of only child were significantly higher than those of non-only child and the scores of individuals from two-parent family were significantly higher than those of a single parent family. However, there was no significant difference between genders, consistent with prior studies (44, 45). This finding may be because family relationships influence a child’s personality traits and cognitive style, with only children often being overcontrolled and protected by their parents (13, 46, 47). This overprotection can have a lasting impact on cognition and increase the risk of disorders through mechanisms, such as heightened susceptibility to stress, which raises the possibility of AjD. Prior research has found similar findings, indicating that overprotective parenting can increase the risk of mental disorders such as depression and stress (47, 48).

The scores of negative emotions, rumination, insomnia, and AjD were significantly different among various age groups, and the score of AjD in the age group of 30 to 35-year-olds was significantly higher than that in other age groups. Prior studies have found that the relationship between age and the prevalence of AjD is controversial (5). The current study found that age is an influencing factor for AjD, and the core symptom pattern of AjD is related to various sociodemographic factors such as a lower family budget (6). Among the interviewed young adults, the 31-35 age group felt more competitive pressure. Behind this, the young people in the 31-35 age group bear the responsibility of taking care of the elderly and children in life. Moreover, they have to face the challenge of “35-year-old crisis” in the workplace. With the increase of various pressures from society, family and work, the probability of AjD also increases. Notably, the AjD score exhibited no gender differences, even though there is substantial evidence that women are at a higher risk for mental disorders (49), possibly as a result of differences in study assessment tools and sample sizes. This suggests more attention should be given to the mental health of young individuals aged over 30 or being the only child in family.

Additionally, the current study found that among the factors related to AjD, including anxiety, depression, stress, rumination, and insomnia, the correlations between each factor were very high (0.445–0.954), consistent with the results of other studies (50). Negative life events, as psychological and social stressors, are significant causes of psychological problems that can easily induce anxiety, depression, and other negative emotional issues. Stress-induced rumination exacerbates anxiety and depression (51). Prior studies have demonstrated a vicious cycle between ruminative thinking and negative emotions; persistent rumination leads to negative emotional outcomes, while negative emotions trigger negative cognition and intensify rumination, resulting in AjD (52–55). Empirical research has also shown that the role of rumination plays in the maintenance of depression, anxiety, and other negative emotions for those with a propensity for rumination (21, 56). Negative emotions can cause sleep disorders, such as short sleep latency, reduced deep sleep, early waking, and difficulty falling asleep after waking (29, 57). As individual levels of negative emotions increase, engaging in ruminative thinking is more likely, creating a vicious cycle between rumination and negative emotions. Higher levels of negative emotions result in poorer sleep quality. Thus, rumination and insomnia act as bridges between negative emotions and AjD.

Negative emotions, rumination, and adjustment disorders are important mediating factors of psychological stress. The findings of the regression analysis indicated that both negative emotions and rumination had predictive effects on AjD and could explain 47.7% of the variance in AjD. Negative emotions have a significant predictive effect on AjD, which has been supported by numerous studies (16) The stress-response syndrome model (58) posits that disorders caused by psychosocial stressors follow a specific developmental pattern, with generally stressful events eliciting intense emotional responses (58), followed by denial of the new reality and a period of re-experience and invasion as the individual recognizes the stressor and all its consequences. Rumination partially mediates the relationship between negative emotions and AjD (59); that is, the more severe the rumination, the more likely it is to produce AjD, which is consistent with prior studies (51). According to Nolen-Hoeksema’s reaction style theory (20), individuals with a ruminative response style tend to repeatedly focus on their negative emotions following a stressful event, engaging in habitual overthinking and introspection, becoming immersed in feelings of sadness and feeling unable to disengage, thereby intensifying the negative emotions triggered by the stressor and exacerbating AjD. This underscores that negative emotions and rumination are significant risk factors for AjD. Interestingly, the results of the regression analysis indicated that depression, anxiety, and stress positively predicted AjD within the dimension of negative emotions in Model 1, whereas the relationship between anxiety and AjD was not significant in Model 3. This suggests that compared to anxiety, depression and stress are more internally focused dimensions whose negative impacts are more pronounced.

Sleep is an indispensable physiological process in humans. Good sleep has beneficial effects, such as eliminating fatigue, storing energy, enhancing immunity, and preventing diseases (60). According to the cognitive model of insomnia, rumination often induces cognitive impairment in patients. Rumination not only makes it difficult for individuals to conduct rational analysis in the face of stressors but also consumes cognitive resources and increases negative self-concern, which is prone to negative bias, distorted cognition, and unconstructive consequences (54). Prior studies have shown that an increase in rumination is associated with longer sleep onset latency and lower sleep quality and efficiency (37). According to the micro-analysis theory of insomnia, the primary mechanism causing insomnia is the excessive activation of physiology, cognition, and emotional systems; ruminative thinking, as an invasive thought, can over-activate the sympathetic nervous system (29), resulting in excessive physiological awakening. Patients with insomnia repeatedly consider their sleep conditions and possible adverse consequences, further causing excessive awakening at the cognitive level (61). The more individuals utilize ruminative thinking, the more their negative emotion level increases; the higher the level of negative emotion, the worse the quality of sleep. Thus, rumination acts as a bridge between negative emotions and insomnia.

According to Morin ‘s comprehensive model (17), insomnia is the result of a dynamic interaction between cognitive dysfunction, maladaptive behaviors (or habits), concerns about the consequences of sleep deprivation (mood, fatigue, and performance), and arousal (mood, cognition, and physiology). Cognitive models (62) provide strong predictions for the development of mental disorders based on the concept of cognitive, emotional, and behavioral interactions, in which maladaptive thoughts lead to negative emotions that produce behavioral changes. Several studies support the role of cognitive dysfunction in insomnia (32, 38). Specifically, individuals with rumination tendencies tend to worry too much about sleep quality, triggering autonomic arousal and emotional distress or anxiety, which leads to distorted perceptions of insomnia, producing maladaptive behaviors such as adaptation disorders.

Additionally, the structural equation model of multiple mediating effects indicated that negative emotions not only directly affected the AjD of young individuals but also indirectly impacted their AjD through rumination and insomnia. In this study, negative emotion could positively predict AjD (β = 0.328, *p* < 0.01), with a direct effect of 0.37. The mediating effects test showed that rumination and insomnia explained 61.9% of the total effect and played a partial mediating role in the relationship between negative emotions and AjD. Rumination can not only exacerbate the vicious circle between negative emotions and negative cognition, resulting in reduced problem-solving ability (63), but it also weakens the emotional regulation ability of young individuals (64), therefore increasing susceptibility to AjD. Despite emerging evidence that AjD is a gateway to more serious mental illnesses, it is important to emphasize that AjD itself is associated with significantly negative outcomes. When young individuals encounter life stress events, they produce negative emotions, which make it relatively easier to generate ruminative thinking, resulting in insomnia and other problems, in addition to eventually leading to adaptation disorders. Specifically, the greater the stress intensity of young individuals, the more likely they are to be immersed in depression, anxiety, pressure, and other negative emotions, thereby inducing ruminative thinking, compulsive thinking, and reflection on negative moods and emotions brought about by life events (65). This negative way of thinking results in insomnia, an incapacity to adapt to changes in the life environment, cognitive distortion, and an inability to deal with problems rationally and calmly, all of which ultimately lead to adaptation disorders (66).

Therefore, alleviating the rumination of young individuals and enhancing their ability to face negative emotions caused by stressful events can improve mental health and reduce the possibility of AjD. This has positive implications for the prevention and intervention of adaptation disorders in young individuals. For those experiencing negative emotions, active intervention should be carried out as soon as possible, focusing on promoting positive and optimistic cognitive tendencies while preventing ruminative thinking and depression; such interventions can also enhance sleep quality and thereby mental health, reducing the possibility of adaptation disorders. This study’s findings can guide effective interventions for adaptation disorders and have positive implications for enhancing young adults’ mental health.

This study has several important limitations. First, it utilized a self-rating scale, which may introduce self-report bias. Future research could enhance assessment accuracy by incorporating structured interview questionnaires or a combination of both methods. Second, this study examined the development of AjD from a cross-sectional perspective. Longitudinal tracking would be beneficial to explore the causal relationships between various variables of AjD and other influencing factors. Additionally, the explanatory power of rumination and insomnia is limited, as negative emotions can lead to diverse reactions that impact social functioning. Future studies should comprehensively consider multiple factors influencing AjD and the interrelationships among these factors.

## 5 Conclusion

In summary, negative emotions, rumination, insomnia, and AjD are interrelated. Rumination and insomnia partially mediate the relationship between negative emotions and AjD. Negative emotions not only directly impact AjD in young adults but also indirectly influence them through rumination and insomnia.

## Data Availability

The raw data supporting the conclusions of this article will be made available by the authors, without undue reservation.
